# Adhesion of *Campylobacter* *jejuni* Is Increased in Association with Foodborne Bacteria

**DOI:** 10.3390/microorganisms8020201

**Published:** 2020-01-31

**Authors:** Anja Klančnik, Ivana Gobin, Barbara Jeršek, Sonja Smole Možina, Darinka Vučković, Magda Tušek Žnidarič, Maja Abram

**Affiliations:** 1 Department of Food Science and Technology, Biotechnical Faculty, University of Ljubljana, Jamnikarjeva 101, SI-1000 Ljubljana, Slovenia; barbka.jersek@bf.uni-lj.si (B.J.); Sonja.Smole@bf.uni-lj.si (S.S.M.); 2 Department of Microbiology, Faculty of Medicine, University of Rijeka, Braće Branchetta 20/1, HR-51000 Rijeka, Croatia; ivanagobin@yahoo.co.uk (I.G.); darinka.vuckovic@medri.uniri.hr (D.V.); maja.abram@medri.uniri.hr (M.A.); 3 Department of Biotechnology and Systems Biology, National Institute of Biology, Večna pot 111, SI-1000 Ljubljana, Slovenia; Magda.Tusek.Znidaric@nib.si; 4 Department of Clinical Microbiology, Clinical Hospital Centre Rijeka, Krešimirova 42, HR-51000 Rijeka, Croatia

**Keywords:** adhesion, abiotic/biotic surface, *Campylobacter jejuni*, co-culture, survival, pathogenicity

## Abstract

The aim of this study was to evaluate *Campylobacter jejuni* NTCT 11168 adhesion to abiotic and biotic surfaces when grown in co-culture with *Escherichia coli* ATCC 11229 and/or *Listeria monocytogenes* 4b. Adhesion of *C. jejuni* to polystyrene and to Caco-2 cells and *Acanthamoeba castellanii* was lower for at least 3 log CFU/mL compared to *E. coli* and *L. monocytogenes*. Electron micrographs of ultrathin sections revealed interactions of *C. jejuni* with host cells. In co-culture with *E. coli* and *L. monocytogenes*, adhesion of *C. jejuni* to all tested surfaces was significantly increased for more than 1 log CFU/mL. There was 10% higher aggregation for *C. jejuni* than for other pathogens, and high co-aggregation of co-cultures of *C. jejuni* with *E. coli* and *L. monocytogenes*. These data show that *C. jejuni* in co-cultures with *E. coli* and *L. monocytogenes* present significantly higher risk than *C. jejuni* as mono-cultures, which need to be taken into account in risk evaluation. *C. jejuni* adhesion is a prerequisite for their colonization, biofilm formation, and further contamination of the environment. *C. jejuni* survival under adverse conditions as a factor in their pathogenicity and depends on their adhesion to different surfaces, not only as individual strains, but also in co-cultures with other bacteria like *E. coli* and *L. monocytogenes*.

## 1. Introduction

According to the World Health Organisation, the incidence and prevalence of campylobacteriosis is still increasing in both developed and developing countries, which is mainly associated with the consumption of undercooked poultry meat products, and with outbreaks arising from contaminated water [1]. The attachment of *Campylobacter jejuni* to materials used as industrial surfaces is an important step for bacterial adaptation, high frequency of resistance, and induced stress response mechanisms. This promotes survival and persistence of *C. jejuni* in the food chain, and can lead to cross-contamination and transmission to a subsequent host [1,2,3,4].

It is known that *C. jejuni* can adhere to different inert surfaces (e.g., stainless steel, glass fibre, beads and coverslips, nitrocellulose membranes, various plastics) [2,3,5,6] and living surfaces (e.g., animal and human intestinal cell lines) [5,6,7,8,9,10]. Cell adhesion to these surfaces is followed by biofilm formation, as an advanced protection mechanism against environmental stresses, antimicrobial agents, and the host immune system. *Campylobacter* adhesion thus contributes to the biofilms or biofouling formations that provide protected modes for bacterial growth and allow bacterial survival in hostile environments, whereby their physiology and behaviour are significantly different from their planktonic counterparts [1,3,11].

Adhesion of *Campylobacter* to epithelial cells represents a very important virulence property in human/ animal infections, and this is crucial not only for *Campylobacter* survival out of a host, but also during host–pathogen interactions, invasion of the intracellular space, and crossing of host barriers, and, thus, for the survival of these bacteria [12]. Contamination by multiple pathogen bacteria, including *Escherichia coli* and *Listeria monocytogenes,* may cause symptoms that are more complex and serious than those caused by infection by only one pathogen [13]. Additionally, to survive environmental stresses, the *Campylobacters* response mechanisms include cell adhesion and invasion, and intracellular survival in protozoa present in water systems, which are also vectors for *Campylobacter* transmission and human infection [12]. *C. jejuni* can thus attach to and survive within free-living protozoa [12,14], which can also occur in the same food-related environments [15]. While harbouring bacteria, free-living protozoa simultaneously serve as their shelter, reservoir, and vector in the environment [16]. However, in addition to protozoa, *C. jejuni* encounter a variety of different bacteria in the environmental niches that they share, with which they inevitably interact. Indeed, today it is widely accepted that multi-species biofilms are the main forms of microbial existence in nature. The development, structure, and function of such polymicrobial communities will depend on the synergistic and antagonistic relationships between the different species [17].

Thus, adhesion of *C. jejuni* to different surfaces and to other microorganisms has a crucial role in their survival and persistence in the food production chain [1,2,3,4], as well as in their pathogenicity and virulence [1,9,18,19,20].

However, the precise molecular mechanisms involved in the multifactorial event of adhesion to abiotic/ biotic surfaces have not been adequately investigated to date. The main cell properties involved in this adhesion include morphology, membranes, membrane proteins, host proteins, fatty acid metabolism, intracellular transport, chaperones, motility, hydrophobicity, quorum-sensing, chemotaxis, aggregation, stress responses, and extracellular polymeric matrix formation. The further external factors involved in cell adhesion include the material surface characteristics and physicochemical factors, and the environmental conditions [2,17,18,20]. The *C. jejuni* binding mechanisms to biotic surfaces generally involve very specific sub-molecular interactions, many of which are not well understood and are strain dependent, such as transmission, capsule production, hydrated extracellular polymeric substances, protein glycosylation, adhesion, epithelial cell invasion, and intestinal colonization and infection [2,3,9,11]. 

The aim of the present study was to investigate *C. jejuni* adhesion in co-cultures with *E. coli* and *L. monocytogenes* to an abiotic polystyrene surface and to the biotic surfaces of human intestinal epithelial Caco-2 cells and *Acanthamoeba castellanii* amoebae*. L. monocytogenes* and *E. coli* were chosen as Gram-positive and Gram-negative food-borne bacteria, respectively, that might have different effects on *C. jejuni* adhesion in co-cultures. The further studies here focused on *C. jejuni* aggregation properties and visualisation of *C. jejuni* interactions with Caco-2 cells and *A. castellanii*.

## 2. Materials and Methods

### 2.1. Bacterial Strains and Growth Conditions

*Campylobacter jejuni* NTCT 11168, *Escherichia coli* ATCC 11229, and *Listeria monocytogenes* (4b, Institute for Hygiene and Microbiology, Wuerzburg, Germany) were used from the culture collection of the Laboratory for Food Microbiology at the Department of Food Science and Technology, Biotechnical Faculty, University of Ljubljana (Ljubljana, Slovenia). *E. coli* and *L. monocytogenes* were grown aerobically in 5 mL Mueller-Hinton broth (MHB; Oxoid, Basingstoke, UK) at 37 °C for 20 h, with continuous shaking at 75 rpm. *C. jejuni* were grown under microaerophilic conditions (5% O_2_, 10% CO_2_, 85% N_2_) in 5 mL MHB with 5% horse blood (Oxoid Basingstoke, UK), at 42 °C for 20 h. 

Bacterial numbers were determined by spectrophotometric measurements of absorbance at 600 nm, and the inocula were prepared in MHB at 10^8^ CFU/mL of each bacterium. For bacterial counting in mono-culture experiments, *C. jejuni, E. coli,* and *L. monocytogenes* were plated on Mueller–Hinton agar (MHA; Oxoid, Basingstoke, UK) plates and incubated under the appropriate conditions (as described above), with the colonies counted and expressed as numbers of bacteria (CFU/mL). For the co-cultures, bacterial cocktails were prepared from 20-h mono-cultures of *C. jejuni*, *E. coli*, and *L. monocytogenes*, mixed in the ratio 1:1:1. For bacterial counting in the co-cultures, the bacterial cocktails were plated on selective agar, as follows: For *C. jejuni*, *Campylobacter* blood-free-medium base (Karmali, CM739; Oxoid) and *Campylobacter* selective supplement (SR0167E; Oxoid); for *E. coli*, Tryptone Bile Agar with X-glucuronide (TBX, 5121562; Biolife) plates; for *L. monocytogenes*, Agar Listeria Ottavani & Agosti (ALOA, 4016052; Biolife, Milan, Italy) plates with ALOA selective supplement (42501; Biolife). The plates were incubated under the appropriate conditions (as described above) and the colonies were counted and expressed as numbers of bacteria (CFU/mL).

### 2.2. Caco-2 Cell Culture Conditions

Caco-2 human intestinal cells were grown in Dulbecco’s minimum essential medium (DMEM; Lonza Group Ltd. Verviers, Verviers, Belgium) supplemented with 10% foetal bovine serum (FBS; Capricorn Scientific GmbH, Ebsdorfergrund, Germany), 2 mM l-glutamine (Lonza Group Ltd. Verviers), 10,000 U/mL penicillin (Lonza Group Ltd. Verviers), and 10,000 mg/mL streptomycin (Lonza Group Ltd. Verviers). The cells were grown routinely in tissue culture flasks (TPP, Trasadingen, Switzerland) at 37 °C in a 5% CO_2_ humidified atmosphere. Cell counts were determined using a Neubauer cell counting chamber (Roth). Confluent cells were harvested by trypsinisation in 0.01% ethylenediaminetetraacetic acid (EDTA, Sigma-Aldrich Chemie GmbH, Taufkirchen, Germany) and maintained in 25-cm^2^ culture flasks (TPP, Trasadingen). Polarized Caco-2 cell monolayers were used after 10 to 14 days of growth.

### 2.3. Acanthamoeba castellanii Growth Conditions

*Acanthamoeba castellanii* ATCC 30234 were maintained axenically in 25-cm^2^ culture flasks (TPP, Trasadingen, Switzerland) with peptone–yeast extract glucose medium (PYG 712 medium: 20 g/L proteose peptone (Oxoid Basingstoke, UK); 1 g/L yeast extract (Oxoid Basingstoke, UK); 18 g/L glucose; 1 g/L sodium citrate·2H_2_O; 9.8 g/L MgSO_4_·7H_2_O; 4.4 g/L Na_2_HPO_4_·12H_2_O; 3.4 g/L KH_2_PO_4_, 0.2 g/L Fe(NH_4_)2(SO_4_)_2_·6H_2_O (Kemika, Croatia) at 25 °C. The *A. castellanii* cells were transferred into fresh medium once a week. Cell counts were determined using a Neubauer cell-counting chamber (Roth, Karlsruhe, Germany).

### 2.4. Adhesion Assays

The adhesion of *C. jejuni*, *E. coli,* and *L. monocytogenes* was assessed as mono-cultures and co-cultures.

#### 2.4.1. Adhesion of Bacteria to Polystyrene

For bacterial adhesion to polystyrene, 96-well polystyrene microtitre plates (Nunc 266 120 polystyrene plates, non-treated sterile with lid, clear flat bottom, 300 μL per well; Nunc, Denmark) were inoculated with 200 μL bacterial inoculum of *C. jejuni*, *E. coli,* and *L. monocytogenes* as mono-cultures and co-cultures. Control wells were prepared by addition of only MHB. After 24 h incubation, the supernatants containing the non-adhered cells were removed from each well, and the plates were rinsed three times with sterile Ringer solution (Kemika, Zagreb, Croatia). Finally, 200 μl Ringer solution was added to each well, followed by sonication at room temperature for 5 min (28 kHz; 300W; IskraPio, Šentjernej, Slovenia) [5]. For counting of *C. jejuni, E. coli,* and *L. monocytogenes* in mono-cultures, each of these bacteria were plated on non-selective MHA plates to determine CFU/mL. For bacterial counting in co-culture experiments, the cocktails of *C. jejuni, E. coli* and *L. monocytogenes* were plated on Karmali, TBX and ALOA selective plates. After incubation of the plates under the appropriate conditions (as described above), the colonies were counted and expressed as mean numbers of adhered bacteria (CFU/mL).

#### 2.4.2. Adhesion of Bacteria to Caco-2 Cells

For bacterial adhesion to Caco-2 cells, these cells were seeded into 24-well tissue culture trays (TPP, Trasadingen) at 10^5^ cells per well for 18 h as previously described [21]. Then, the DMEM supplemented with 10% FBS, 2 mM L-glutamine, 10,000 U/mL penicillin, and 10,000 mg/mL streptomycin was replaced, and the attached cells were washed twice with DMEM supplemented with 10% FBS, without antibiotics. After 30 min, the bacterial inocula with 10^8^ CFU/mL *C. jejuni*, *E. coli,* and *L. monocytogenes* as mono-cultures or co-cultures were added. The multiplicity of infection (MOI) obtained was 1000:1 (bacteria: Caco-2 cells). The plates were centrifuged at 170 × *g* for 5 min, followed by incubation at 37 °C for 2 h. At the end of this incubation, the monolayers were washed twice with DMEM supplemented with 10% FBS without antibiotics, to remove the unbound bacteria. The monolayers were then lysed by the addition of 1 mL 1.0% Triton X-100 (Sigma Aldrich Chemie GmbH, Steinheim, Germany) and sonicated at room temperature for 1 min (40 kHz, 200 W; Bacto Sonic Bandelin, Berlin, Germany). The intracellular and adhered bacteria released were counted using MHA plates, and were expressed as the total numbers of bacteria. The differences between the total numbers of bacteria and the numbers of intracellular bacteria were calculated to obtain the numbers of adherent bacterial cells. For the measurements of intracellular bacteria, DMEM supplemented with 10% FBS containing 100 μg/mL gentamicin was added for 1 h, to kill the extracellular bacteria. Then the antibiotic was removed by washing twice with tissue culture medium, and the cells were lysed with the addition of 1 mL 1.0% Triton X-100, with sonication at room temperature for 1 min (40 kHz, 200 W). The intracellular bacteria released were counted on MHA plates, and are expressed as mean numbers of intracellular bacteria (CFU/mL). For the counting of *C. jejuni*, *E. coli*, and *L. monocytogenes*, the procedures were the same as described in the section below: Adhesion of bacteria to *A. castellanii*.

#### 2.4.3. Adhesion of Bacteria to *A. castellanii*

For bacterial adhesion to *A. castellanii*, the *A. castellanii* were seeded in PYG 712 medium in 24-well tissue culture trays (TPP, Trasadingen) at 10^5^ cells per well and left at 25 °C for 2 h, as previously described [22]. Then, the medium was replaced and the attached *A. castellanii* cells were washed twice with Page’s Amoeba Saline (PAS; 0.12 g/L NaCl, 2.5 mg/L MgSO_4_·2H_2_O, 4 5 mg/L CaCl_2_·2H_2_O, 358 5 mg/L Na_2_HPO_4_·12 H_2_O, 136 5 mg/L KH_2_PO_4_; Kemika, Croatia). After 30 min, the bacterial inoculum with 10^8^ CFU/mL *C. jejuni*, *E. coli,* and *L. monocytogenes* as mono-cultures or as co-cultures were added. The multiplicity of infection (MOI) obtained was 1000:1 (bacteria:*A. castellanii*). The plates were centrifuged at 170× *g* for 5 min, and then incubated at 25 °C for 2 h. After the incubation, the non-adherent bacteria were removed by washing the wells twice with PAS. 

The differences between the total numbers of bacteria and the numbers of intracellular bacteria were calculated to obtain the number of adherent bacterial cells on the *A. castellanii* amoebae surface. For determination of the number of intracellular bacteria, the *A. castellanii* cells (from 3 wells) were re-suspended in 1 mL PAS containing 100 μg/mL gentamicin (Sigma-Aldrich Chemie GmbH, Taufkirchen, Germany) and incubated at room temperature for 1 h. The cell monolayers were then washed twice with PAS, and 1 mL PAS containing 0.3% Triton X-100 (Sigma Aldrich Chemie GmbH, Taufkirchen, Germany) was added, and sonicated for 1 min at room temperature (40 kHz, 200 W; Bacto Sonic Bandelin, Germany), to lyse the amoebae. These samples were plated on MHA plates when mono-cultures were used, and on Karmali, TBX, and ALOA plates when co-cultures were used. After incubation (37 °C, 24–48 h in the appropriate atmosphere), the data were expressed as mean numbers of intracellular bacteria (CFU/mL). The total numbers of (adherent and intracellular) bacteria were determined in the same way as the numbers of intracellular bacteria, obtained without gentamicin treatment.

### 2.5. Aggregation and Co-Aggregation Assays

The aggregation and co-aggregation assays were performed according to Kos et al. with some modifications. *C. jejuni*, *E. coli*, and *L. monocytogenes* were grown on agar plates, on Karmali *Campylobacter* agar, TBX and ALOA, respectively [23]. The bacterial cells were harvested and washed twice with sterile phosphate-buffered saline (Oxoid, Basingstoke, UK), with centrifugation at 1075× *g* for 5 min. The pellets were resuspended in phosphate-buffered saline, and the optical densities (OD_600_) of the bacterial suspensions were determined using a microplate reader (Tecan Group AG, Männedorf, Switzerland). The bacterial suspensions were then readjusted with sterile phosphate-buffered saline to an OD_600_ of 1 (*C. jejuni*: about 0.5 × 10^9^ CFU/mL; *E. coli* and *L. monocytogenes*: 1 × 10^9^ CFU/mL).

Bacterial suspensions for aggregation assays were divided into glass test-tubes (4 mL) and mixed by vortexing. The OD_600_ of the carefully pipetted supernatants (upper layers) were measured immediately (0 h), and then after 2 h and 24 h of incubation at room temperature. The aggregation percentages were determined according to Equation (1).

(1) $$\%\;aggregation=\left(1-\frac{OD_{Time}}{OD_{T0}}\right)\times 100$$

where *OD_Time_* is the *OD*_600_ at the specified times (2 h, 24 h), with *OD_T0_* at time zero (t = 0 h).

Bacterial suspensions for the co-aggregation assays were prepared in the same way as for the aggregation assays. Equal volumes (2 mL) of each bacterial suspension were mixed with the other bacterial suspensions, as required (i.e., *C. jejuni* plus *E. coli*; *C. jejuni* plus *L. monocytogenes; E. coli* plus *L. monocytogenes*) in glass test-tubes. The OD_600_ of the carefully pipetted supernatants (upper layers) were measured immediately (0 h), and after 2 h and 24 h. The co-aggregation percentages were determined using Equation (2).

(2) $$\%\;co-aggregation=\;\left[1-\frac{OD_{Mix}}{\frac{OD_{Bac1}+OD_{Bac2}}{2}}\right]\times 100$$

where *OD_Mix_* is the *OD*_600_ of the co-cultures (as indicated above) and *OD_Bac_* is the *OD*_600_ of the bacterial mono-cultures (i.e., *C. jejuni*, *E. coli*, *L. monocytogenes*) at the specified times (2 h, 24 h). 

### 2.6. Transmission Electron Microscopy

*Campylobacter jejuni* cells adhered to Caco-2 cells and *A. castellanii* were examined using transmission electron microscopy (TEM). Caco-2 cells and *A. castellanii* were grown in 24-well tissue culture trays, and the *C. jejuni*, *E. coli* and *L. monocytogenes* as mono-cultures or as co-cultures were added as described above (*Adhesion assays*), and incubated for 2 h. After this incubation, the non-adherent bacteria were removed by washing twice with DMEM (Caco-2 cells) and with PAS (*A. castellanii*), and the cells were immediately fixed with 2.5% glutaraldehyde (SPI, West Chester, PA, USA) in 0.1 M phosphate buffer. The fixed cells were scraped, post-fixed with 1% OsO_4_ (SPI, West Chester, PA, USA), and embedded in Agar 100 resin (Agar Scientific, London, UK). Ultrathin sections were stained with 1% water solution of uranyl acetate (SPI, West Chester, PA, USA) and lead citrate (SPI, West Chester, PA, USA).

The aggregated and co-aggregated samples were visualized using TEM. These were prepared with the negative staining method reported previously [7]. Grids were examined using an electron microscope (Philips CM 100; Philips Electronics N.V. Eindhoven, The Netherlands), which was operated at 80 kV. The images were recorded with a CCD camera (Bioscan) or a digital camera (Orius SC 200) using the Digital Micrograph Software (Gatan Inc., Washington, DC, USA). 

### 2.7. Statistical Analysis

The statistical analysis was performed using SPSS Statistics version 20 (IBM Corp., Armonk, NY, USA). The data for the adhesion properties are presented as the number of bacteria (CFU/mL) and the data for aggregation are presented as percentages of aggregation and co-aggregation. The significances of the differences between the different bacteria as mono-cultures and co-cultures were determined using one-way analysis of variance (ANOVA), and are considered significant at *p* <0.05. All of the experiments were carried out in at least three replicas. 

## 3. Results

### 3.1. Adhesion 

**Figure 1:**
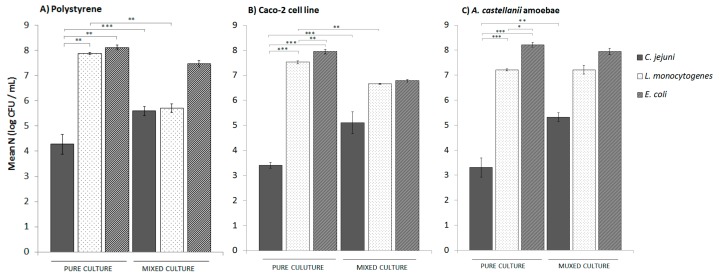
Adhesion of *C. jejuni*, *E. coli,* and *L. monocytogenes* as mono-cultures and as mixed co-cultures of all three of these bacteria to polystyrene, co-incubation for 24 h (**A**); Caco-2 cells, co-incubation for 2 h (**B**); and *A. castellanii* amoebae, co-incubation for 2 h (**C**). Data are expressed as means ± standard deviation. *, *p* < 0.05; **, *p* < 0.01; ***, *p* < 0.001; as indicated.

For the adhesion of *C. jejuni*, polystyrene was used as the abiotic surface, and Caco-2 cells and *A. castellanii* as the biotic surfaces. Adhesion to polystyrene (Figure 1) was determined after 24 h, as reported previously [11], and to Caco-2 cells and *A. castellanii* after 2 h, to avoid the main bacterial internalisation and the formation of amoebal cysts [24]. The numbers of intracellular bacteria were monitored for the Caco-2 cells and *A. castellanii*, and these levels were taken into account when calculating the numbers of adhered cells. In the 2 h of incubation, the numbers of intracellular bacteria were low, with >99% of the *C. jejuni, E. coli,* and *L. monocytogenes* attached to the Caco-2 cells and to *A. castellanii* (data not shown). 

The level of adhesion of *C. jejuni* to polystyrene was 4.28 ± 0.38 log CFU/mL, while this was lower for both of the biotic surfaces, as 3.40 ± 0.11 log CFU/mL and 3.31 ± 0.10 log CFU/mL for the *C. jejuni* cells adhered to the Caco-2 cells and *A. castellanii*, respectively (Figure 1). On the other hand, the adhesion levels of *E. coli* and *L. monocytogenes* were comparable, and were significantly greater than for *C. jejuni*, from 7.22 ± 0.02 log CFU/mL to 8.21 ± 0.18 log CFU/mL across the three surfaces tested (i.e., polystyrene, Caco-2 cells, amoeba cells). When in co-culture with *E. coli* and *L. monocytogenes*, the levels of adhered *C. jejuni* cells were significantly increased by up to 2 log CFU/mL in comparison to the *C. jejuni* mono-cultures, and this was not dependent on the type of surface (Figure 1).

In contrast, the levels of *E. coli* and *L. monocytogenes* cells in co-cultures that were adhered to polystyrene and Caco-2 cells were 0.5 to 2.5 log CFU/mL lower than when they were in mono-cultures. There were no significant differences in the adhesion of *E. coli* and *L. monocytogenes* to *A. castellanii* as mono-cultures and as co-cultures.

**Figure 2:**
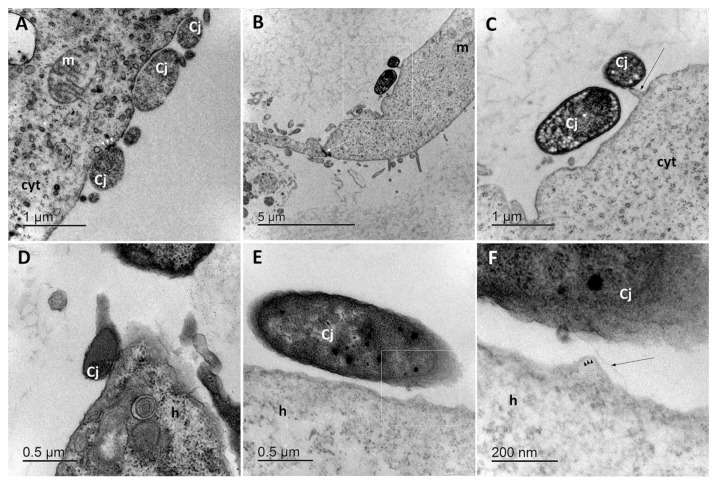
Representative transmission electron micrographs of *C. jejuni* cells adhered to biotic surfaces. (**A**–**C**) *Adhesion* to Caco-2 cells, with enlargement of the box in (**B**) shown in (**C**). (**D**–**F**) *Adhesion* to *A. castellanii*, with enlargement of the box in (**D**) shown in (**F**). Cj, *Campylobacter jejuni*; m, mitochondrion; cyt, cytoplasm of Caco-2 cell; h, hyaloplasm of *A. castellanii*. Scale bars, as shown.

The *C. jejuni* that adhered to Caco-2 cells and *A. castellani* were examined using TEM. This showed that after 2 h of co-cultivation, *C. jejuni* cells were attached to the surfaces of Caco-2 cells (Figure 2A) and *A. castellani* (Figure 2D). Closer examination of the TEM micrographs revealed the interactions of *C. jejuni* with the host cells. The Caco-2 cells in the monolayers were tightly connected through many cell-to-cell junctions (Figure 2B, white arrow). Extensions on the surface of the Caco-2 cells (Figure 2C, black arrow) indicated the beginnings of the adhesion of *C. jejuni*. When contact between these bacteria and the cell surface was established, electron dense inclusions appeared just below the plasma membranes of the Caco-2 cells (Figure 2A, white arrowheads). Similar to this projection on the Caco-2 cells, bulges formed on the surface of the *A. castellanii* (Figure 2F, black arrowheads). Actin microfilaments in *Acanthamoeba* are located and most concentrated just beneath the plasma membrane and are responsible for cytoplasmic protrusions [14,15]. This formation of bulges (Figure 2F) thus appeared to be part of the *C. jejuni*–*A. castellanii* interactions that led to adhesion. Occasionally, the interactions between the bacteria and the cell surface were seen as tiny filamentous structures (Figure 2F, black arrow). As these *C. jejuni* interactions with the Caco-2 cells and *Acanthamoeba* are very complex, further studies of *C. jejuni* in co-cultures with other bacteria are necessary. 

### 3.2. Aggregation

**Figure 3:**
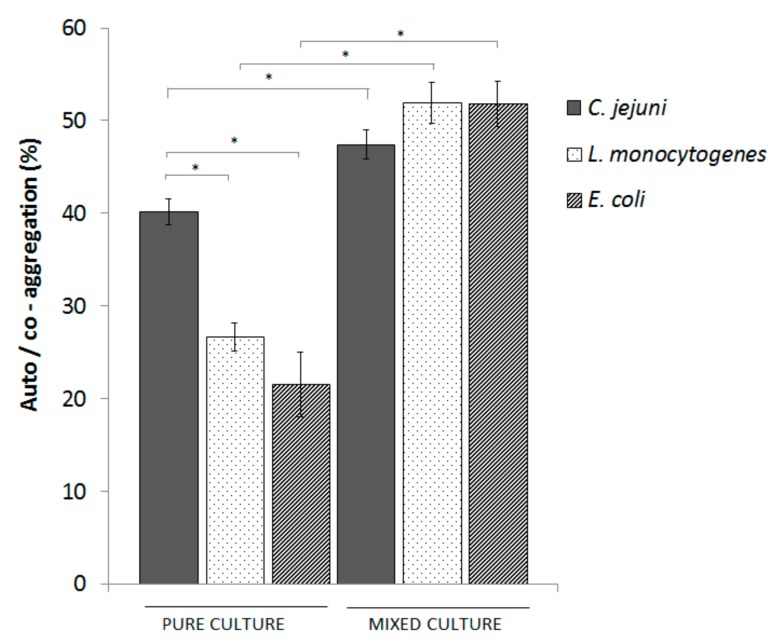
Aggregation and co-aggregation of *C. jejuni*, *E. coli,* and *L. monocytogenes* as mono-cultures and mixed co-culture of the three bacteria after 24 h. Data are means ± standard deviations. *, *p* < 0.001; as indicated.

The aggregation of individual *C. jejuni, E. coli* and *L. monocytogenes* cells and their co-aggregation were evaluated after 2 h and 24 h. The aggregation of all of the bacteria tested was <7% in the first 2 h (results not shown), but increased significantly after 24 h, up to 40.1% ± 1.4% for *C. jejuni*, 21.5% ±3.8% for *E. coli*, and 26.6% ± 1.5% for *L. monocytogenes* (Figure 3). The co-aggregation of the mixed *C. jejuni*, *E. coli,* and *L. monocytogenes* was 69.9%, which showed enhanced aggregation of all three bacteria (*C. jejuni*, 47.1%; *E. coli*, 51.8%; *L. monocytogenes*, 51.9%), in comparison to the individual cultures.

**Figure 4:**
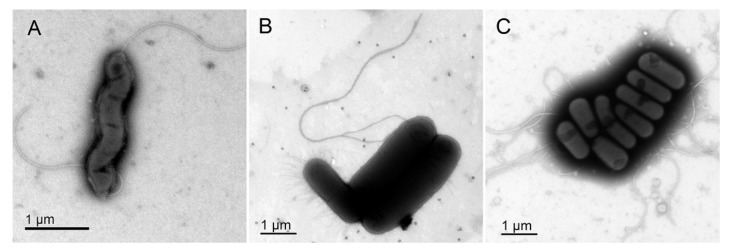
Representative transmission electron micrographs of the negatively stained control bacteria. (**A**) *C. jejuni.* (**B**) *E. coli*. (**C**) *L. monocytogenes.* Scale bars, as shown.

**Figure 5:**
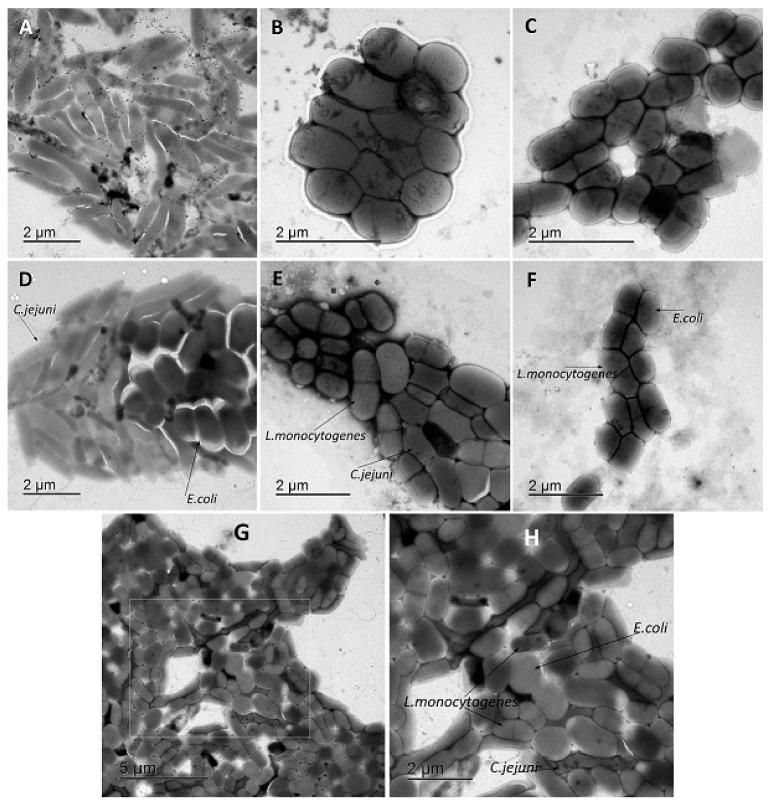
Representative transmission electron micrographs of the bacterial aggregation and co-aggregation. (**A**) *C. jejuni*. (**B**) *E. coli*. (**C**) *L. monocytogenes*. (**D**) Mixed culture of *C. jejuni* and *E. coli*. (**E**) Mixed culture of *C. jejuni* and *L. monocytogenes*. (**F**) Mixed culture of *E. coli* and *L. monocytogenes*. (**G**) Mixed culture of *C. jejuni*, *E. coli* and *L. monocytogenes*. (**H**) Enlargement of box shown in (**G**). Scale bars, as shown.

Representative transmission electron micrographs (TEM) in Figure 4 show the morphology of control *C. jejuni*, *E. coli,* and *L. monocytogenes* planktonic cells. Aggregates of *C. jejuni*, *E. coli,* and *L. monocytogenes* are shown in Figure 5A–C, where these TEM micrographs show the elongated *C. jejuni* cells (Figure 5A), the short coccoid bacilli shape of *E. coli* cells that were more closely packed than for *C. jejuni* (Figure 5B), and the mono or diplo-bacilli of *L. monocytogenes* (Figure 5C). Aggregates of *C. jejuni* and *E. coli* and of *C. jejuni* and *L. monocytogenes* co-cultures were also larger and included more bacterial cells than for the aggregates of *E. coli* and *L. monocytogenes* co-cultures. Then, when all three of these bacteria were included in co-cultures together, they were closely aggregated, and organized such that the *E. coli* and *L. monocytogenes* cells were arranged on the inside of the aggregates, and the *C. jejuni* cells formed the edges of the aggregates (Figure 5G,H). In these co-cultures of all three bacteria, the *C. jejuni* cells were more elongated (Figure 5G,H) than in the *C. jejuni* mono-cultures (Figure 5A).

## 4. Discussion

Although there remain high levels of *Campylobacter* infections, these have not been brought under control and have instead showed significant increase over recent years [1,25]. At the same time, the mechanism by which *C. jejuni* causes human disease is still poorly understood [26]. As bacterial adhesion is important for biofilm formation, we investigated the modulation of *C. jejuni* adhesion in co-cultures of the foodborne pathogens *L. monocytogenes* and *E. coli*. We asked whether *E. coli* and *L. monocytogenes* can influence the levels of *C. jejuni* adherence to different surfaces, such as those of foods, hosts, or environments, which were here exemplified by the surfaces of polystyrene, human intestinal epithelial cells (Caco-2 cells) and *A. castellanii* amoebae. Adhesion to polystyrene was determined after 24 h [11], and to Caco-2 cells and *A. castellanii* after 2 h, to avoid the main bacterial internalization and the formation of amoebal cysts [24]. In our experiments, we confirmed that these were the optimal incubation times and therefore we consider that the results are comparable [5,22].

Adhesion of bacteria to abiotic surfaces is mediated by non-specific (e.g., hydrophobic) interactions [27], whereas their adhesion to living cells is accomplished through specific molecular mechanisms, such as those involving lectin, specific ligands, or adhesion [3,9,11,28].

The adhesion of *C. jejuni* was similar to that previously observed [27], although *Campylobacter* biofilms, biomass, and ultrastructure differ between strains [29]. In the present study, the adhesion of *C. jejuni* to polystyrene was higher than seen for adhesion to the Caco-2 cells and *A. castellanii*. This attachment of *C. jejuni* cells to the biotic surfaces was lower because of the host-cell defences, such as the mucus layer that forms a barrier and can limit access of microbes to the epithelial cell surface, and the peristalsis that mechanically removes unattached bacteria [2,26].

Lower adhesion to the eukaryotic cell host also directly influences the number of successfully internalized bacterial. Internalisation of *C. jejuni* in co-cultures with amoebae depends on type of amoeba and the bacterial viability [15]. Oloffson et al. showed that approximately 90% of viable *C. jejuni* cells were attached to *A. polyphaga*, with 10% in the intracellular vacuoles of these amoebae after 1 h of incubation [15]. Prolonged incubations (24–96 h) resulted in more than 80% of the *C. jejuni* inside these intracellular vacuoles. Vieira *et al.* described the contribution of a *Campylobacter* efflux pump to bacterial survival within amoebae [29]. In the present study with *A. castellanii*, <0.01% of the viable *C. jejuni* cells were internalized after 2 h of incubation with *A. castellanii*. As for adhesion, the internalization of *C. jejuni* into the Caco-2 cells in the present study was also comparable to published data [28].

Additionally, strain differences and stress responses to environmental conditions such as temperature (i.e., 25 °C, 37 °C) and oxygen concentration (i.e., aerobic, microaerophilic) can affect the *C. jejuni* interactions with *A. castellanii*, and consequently modulate *C. jejuni* viability, adhesion and internalisation [14]. *C. jejuni* is a microaerophilic organism that is sensitive to atmospheric oxygen, while variable oxygen concentrations will be encountered within cell lines and various hosts [2]. Thus, as expected, the adhesion of *C. jejuni* at 25 °C and 37 °C and under the aerobic conditions of the present study was lower when compared to that of *E. coli* and *L. monocytogenes*, which showed higher adhesion regardless of the target surface used. This is in consistence with the well-studied formation of *E. coli* and *L. monocytogenes* mono-culture biofilms, where the initial adhesion occurs within a few seconds [30]. This thus contributes to their persistence on food-processing surfaces and the subsequent cross-contamination or re-contamination of food products [30].

For *L. monocytogenes*, Doyscher et al. showed that these bacteria can form ‘backpacks’ on the surface of *A. castellanii* within the first 10 min to 20 min of exposure, and then these amoebae start to phagocytose entire aggregates that are attached to their surface [31]. Then after approximately 6 h, all of these backpacks are phagocytosed and the *L. monocytogenes* cells are digested in the food vacuoles of *A. castellanii*. We showed here that also after 2 h approximately 10^4^ and 10^3^ CFU/mL *L. monocytogenes* cells were intracellular in the *A. castellanii* when these *L. monocytogenes* were used as mono-cultures and as co-cultures with *C. jejuni* and *E. coli*, respectively. The lipopolysaccharides on the surface of Gram-negative bacteria have a major role in *E. coli* uptake by *A. castellani* [32]. The present study, a non-pathogenic *E. coli* strain was used (ATCC 11229), and these bacteria were not internalized by *A. castellanii*. It appears that *A. castellanii* represent a good model to study *C. jejuni, E. coli,* and *L. monocytogenes* adhesion under in vivo conditions.

Furthermore, we investigated whether *E. coli* and *L. monocytogenes* can influence *C. jejuni* adherence to the different surfaces. Interestingly, these data showed significantly enhanced *C. jejuni* adhesion to both the abiotic and biotic surfaces when in co-cultures with *E. coli* and *L. monocytogenes*, in comparison to the adhesion of *C. jejuni* mono-cultures. These data constitute an example of the *C. jejuni* survival strategy, and they highlight the modulation of adhesion in co-cultures that is potentially present in water, food products, and food production environments, and in the poultry gut and human digestive tract, with particular concern related to *Campylobacter* outbreaks. However, these pathways are compatible for other food-borne pathogenic bacteria as well, and the shared pathways lead to cross contamination. Studies of bacterial interactions are important, as co-infection with food-borne pathogens like *E. coli* or *L. monocytogenes* can promote *C. jejuni* colonization and infection, although *L. monocytogenes* may also reduce *C. jejuni* colonization [13]. Also, the species composition of biofilms in vivo can change by up to 40% every day with multispecies biofilms [18], where the levels of *C. jejuni* have been shown to be enhanced after 24 h when incubated with other bacteria at two initial inoculum densities [27]. Positive impacts on *C. jejuni* adhesion might be due to its increased growth or increased biomass, to its co-aggregation with other bacteria to form clusters that can deposit on the surfaces to initiate colonization, or to larger amounts of additional extracellular matrices of other species that can provide micro-niches in multispecies biofilms [32,33]. Additionally, the metabolic by-products of one organism might serve to support the growth of another, while the adhesion of one species might provide ligands that promote the attachment of others [34]. The reasons for the increased adhesion of *C. jejuni* might also relate to their survival characteristics, their ability to response to stress conditions, which include oxidative stress using O_2_ as an advantage for biofilm development, or their transition to more resistant cell forms [27,35]. Furthermore, the mechanism of *C. jejuni* biofilm formation might be a general response to adverse conditions that supports their high genetic diversity, which might contribute to longer term adaptation to varying environmental conditions, and the release of eDNA under biofilm conditions, which can promote the conditions that potentially serve for horizontal gene transfer [11,34]. Indeed, co-culture-enhanced *C. jejuni* adhesion can lead to increased resistance and survival of these fastidious and oxygen-sensitive *Campylobacter* in these environments, and under industrial conditions and during host interactions. The following disease is thus defined not only by the host–pathogen relationship, but also by a spectrum of host-microbe pathogenic mechanisms, microbe-microbe interactions, host immunity-mediated antimicrobial defences, and environmental factors [34].

As has been shown, several important foodborne pathogens have interspecies interactions when adhered or when in biofilms [17,27,32]. Furthermore, bacterial aggregation is an important mechanism for foodborne bacterial pathogen attachment to abiotic surfaces and initiation of micro-colony growth, and it represents the main type of cooperative interactions that facilitate co-adhesion of bacterial pairs to the surface [17,34]. In terms of co-aggregation, the succession of bacterial biofilms is tightly controlled by specific cell-surface-associated receptor–ligand interactions, and these often result in enhanced levels of multispecies biofilm formation [17]. In the present study, *C. jejuni* underwent significant aggregates after 24 h, which indicated the potential mechanism of *C. jejuni* for synergistic interactions between different species during biofilm formation. All three investigated bacteria could be found simultaneously in food-producing animals affecting each other. The co-culturing of *C. jejuni* with *E. coli* and *L. monocytogenes* resulted in even higher co-aggregation when compared to *C. jejuni* as mono-cultures. Consequently, we can propose that both *E. coli* and *L. monocytogenes* can serve as pioneering attached bacteria that can be recognized and provide anchors for secondary bacterial colonizers. Additionally, the close contact that results from co-aggregation events facilitates cooperation between the different species, as reported previously [17,36,37]. Co-aggregation of specific secondary colonizers to already attached biofilms affects the organisation of oral multispecies biofilms [32,36,37], although this has not been confirmed for other bacteria. However, the representative TEM micrographs in the present study showed morphological changes of the bacteria tested as aggregates. In the aggregates and co-aggregates, the *C. jejuni* cells were elongated, with enhanced aggregates in co-cultures. Of particular interest, with all three of the bacteria in co-cultures, they formed specifically organized aggregates where *C. jejuni* formed the edges of the aggregates. 

## 5. Conclusions

The data are of particular importance for risk assessment of *Campylobacter* in food. We have shown that studies of *C. jejuni* mono-culture adhesion can lead to misleading conclusions, as the presence of microflora in food and other environments can be decisive for the infective potential of *C. jejuni*. The present data indicate significant modulation of *C. jejuni* adhesion and suggest that bacteria that are commonly present in vivo within environments related to the food industry and in humans can facilitates and accelerate *C. jejuni* adhesion. These data suggest that, in partnership with other foodborne bacteria, *C. jejuni* present significantly higher risk than when in mono-culture because of this enhanced adhesion. Further investigations into *C. jejuni* adhesion properties and the complexity of these interactions within multispecies biofilms are therefore crucial for our understanding of *C. jejuni* adaptation, resistance, and virulence. This should allow for the modification of aspects within food processing environments to reduce microbial persistence, potentially through changed surfaces or the development of effective cleaning programmes, to consequently reduce *Campylobacter* and their infection transmission.
